# Direct Evidence Revealing Structural Elements Essential for the High Binding Ability of Bisphenol A to Human Estrogen-Related Receptor-γ

**DOI:** 10.1289/ehp.10587

**Published:** 2007-10-05

**Authors:** Hiroyuki Okada, Takatoshi Tokunaga, Xiaohui Liu, Sayaka Takayanagi, Ayami Matsushima, Yasuyuki Shimohigashi

**Affiliations:** Laboratory of Structure-Function Biochemistry, Department of Chemistry, The Research-Education Centre of Risk Science, Faculty and Graduate School of Sciences, Kyushu University, Fukuoka, Japan

**Keywords:** bisphenol A, constitutive activity, endocrine disruptor, estrogen receptor, estrogen-related receptor-γ, inverse agonist, nuclear receptor

## Abstract

**Background:**

Various lines of evidence have shown that bisphenol A [BPA; HO-C_6_H_4_-C(CH_3_)_2_-C_6_H_4_-OH] acts as an endocrine disruptor when present in very low doses. We have recently demonstrated that BPA binds strongly to human estrogen-related receptor-γ (ERR-γ ) in a binding assay using [^3^H]4-hydroxytamoxifen ([^3^H]4-OHT). We also demonstrated that BPA inhibits the deactivation activity of 4-OHT.

**Objectives:**

In the present study, we intended to obtain direct evidence that BPA interacts with ERR-γ as a strong binder, and also to clarify the structural requirements of BPA for its binding to ERR-γ.

**Methods:**

We examined [^3^H]BPA in the saturation binding assay using the ligand binding domain of ERR-γ and analyzed the result using Scatchard plot analysis. A number of BPA derivatives were tested in the competitive binding assay using [^3^H]BPA as a tracer and in the luciferase reporter gene assay.

**Results:**

[^3^H]BPA showed a *K*_D_ of 5.50 nM at a *B*_max_ of 14.4 nmol/mg. When we examined BPA derivatives to evaluate the structural essentials required for the binding of BPA to ERR-γ , we found that only one of the two phenol-hydroxyl groups was essential for the full binding. The maximal activity was attained when one of the methyl groups was removed. All of the potent BPA derivatives retained a high constitutive basal activity of ERR-γ in the luciferase reporter gene assay and exhibited a distinct inhibitory activity against 4-OHT.

**Conclusion:**

These results indicate that the phenol derivatives are potent candidates for the endocrine disruptor that binds to ERR-γ.

Bisphenol A [BPA; 2,2-bis(4-hydroxyphenyl)propane] has a symmetrical chemical structure of HO-C_6_H_4_-C(CH_3_)_2_-C_6_H_4_-OH. BPA is used mainly in the production of polycarbonate plastics and epoxy resins. Its worldwide manufacture is approximately 3.2 million metric tons/year. BPA has been acknowledged to be an estrogenic chemical able to interact with human estrogen receptors (ER) (Dodds and Lawson 1938; Krishnan et al. 1993; Olea et al. 1996), and many lines of evidence have revealed that BPA, at even low doses, acts as an endocrine disruptor (Gupta 2000; Nagel et al. 1997; vom Saal et al. 1998; Welshons et al. 2003). However, its binding to and hormonal interaction with ER are extremely weak, 2–3 orders of magnitude lower than those of natural hormones, and thus the intrinsic significance of these low-dose effects is rather intangible and obscure (Safe et al. 2002). These facts led us to hypothesize that BPA may interact with nuclear receptors (NRs) other than ER.

We have recently demonstrated that BPA binds strongly to estrogen-related receptor-γ (ERR-γ ) with high constitutive activity (Takayanagi et al. 2006). ERR-γ is a member of the human NR family and the estrogen-related receptor (ERR) subfamily of orphan NRs, which are closely related to the ERs ER-α and ER-β (Giguère 2002; Horard and Vanacker 2003). The ERR family includes three members—ERR-α , ERR-β , and ERR-γ —with ERR-γ being the most recently identified (Eudy et al. 1998; Hong et al. 1999). The amino acid sequences are quite highly conserved among ERRs and ERs, but 17β-estradiol (E_2_), a natural ligand of ERs, does not bind to any of the ERR family members. Our discovery that BPA binds strongly to ERR-γ , but not to ERs, indicates that the effects of the so-called endocrine disruptors should be examined for all NRs without delay.

ERR-γ is expressed in a tissue-restricted manner—for example, very strongly in the mammalian brain during development, and then in the brain, lung, and many other tissues during adulthood (Eudy et al. 1998; Heard et al. 2000; Lorke et al. 2000). Our preliminary results have shown that the highest expression is brought about in the placenta (Takeda Y, Sumiyoshi M, Liu X, Matsushima A, Shimohigashi M, Shimohigashi Y, unpublished data). Strong binding of BPA to ERR-γ would affect not only the physiologic functions but also the metabolism of this NR as a transcription-activating factor. Although the intrinsic physiologic functions of ERR-γ have not yet been clarified, it is crucial that a structure–function study be performed to clarify the structural requirements for the binding of BPA to ERR-γ.

In a previous study (Takayanagi et al. 2006), we used tritium (^3^H)-labeled 4-hydroxytamoxifen (4-OHT) as a tracer in a receptor binding assay for ERR-γ . 4-OHT binds strongly to ERR-γ and deactivates it as an inverse agonist, decreasing the very high level of spontaneous constitutive activity (Coward et al. 2001). As a substitute for [^3^H]4-OHT, BPA was found to be as potent as 4-OHT in this binding assay. Furthermore, BPA was found to retain or rescue ERR-γ ’s high basal constitutive activity in the reporter gene assay for ERR-γ using HeLa cells. These results indicated that BPA and 4-OHT bind to ERR-γ with equal strength, but have structural differences that affect their occupation of ERR-γ ’s ligand binding pocket. In the complex formed between 4-OHT and the ERR-γ –ligand binding domain (LBD), 4-OHT remained at the ligand binding pocket of ERR-γ –LBD, but the α-helix 12 of the receptor was repositioned from the activation conformation (Greschik et al. 2004; Wang et al. 2006). In contrast, BPA was suggested to bind to the pocket without changing the positioning of helix 12, and thus preserved the high receptor constitutive activity of ERR-γ.

It is evident that the binding ability of BPA to ERR-γ should be examined by means of tritium-labeled BPA. Fortunately, [^3^H]BPA is now commercially available; thus, in the present study we performed the first saturation binding assay for direct exploration of the binding characteristics of BPA. We then established a competitive receptor binding assay in which chemicals were assessed for their ability to displace [^3^H]BPA from the receptor binding pocket. In particular, industrial chemical products of BPA analogs were inspected structurally in order to better understand the structural elements of BPA that are required for binding to the ERR. Here we describe the structural elements of BPA that are required for the binding to ERR-γ –LBD and for maintaining the receptor in an active conformation.

## Materials and Methods

### Chemicals

We purchased 2,2-bis(4-hydroxyphenyl)propane and 4,4-isopropylidene-diphenol, both denoted as BPA, from Tokyo Kasei Kogyo Co. (Tokyo, Japan), Nakarai Tesque (Kyoto, Japan), Aldrich (Madison, WI, USA), Junsei Chemical (Tokyo, Japan), Acros (Geel, Belgium), Lancaster Synthesis (Windham, NH, USA), Merck (Darmstadt, Germany), and Fluka (Buchs, Switzerland). The purity designated on the labels varied from 95 to 99%. We also obtained the following analogs of BPA: bisphenol AF [2,2-bis(4-hydroxyphenyl)hexafluoropropane; Tokyo Kasei], bisphenol AP [4,4′-(1-phenylethylidene)bisphenol; Tokyo Kasei], bisphenol B [2,2-bis(4-hydroxyphenyl)butane; Tokyo Kasei], bisphenol E [2,2-bis(4-hydroxyphenyl)ethane; Aldrich], and bisphenol F [bis(4-hydroxyphenyl)methane; Tokyo Kasei].

4-α-Cumylphenol [2-(4-hydroxyphenyl)-2-phenylpropane], 4-*tert*-amylphenol, 4-*tert*-butylphenol, 4-isopropylphenol, and 4-ethylphenol were obtained from Tokyo Kasei. 2,2-Diphenyl propane, and 4-*tert*-octylphenol were obtained from Aldrich, and *p*-cresol and phenol from Kishida Chemical (Osaka, Japan).

### Preparation of receptor protein GST-fused ERR-γ –LBD

ERR-γ –LBD was amplified from a human kidney cDNA library (Clontech Laboratories, Mountain View, CA, USA) by polymerase chain reaction (PCR) using gene-specific primers and cloned into pGEX6P-1 (Amersham Biosciences, Piscataway, NJ, USA). Glutathione *S*-transferase (GST)–fused receptor protein expressed in *Escherichia coli* BL21α was purified on an affinity column of glutathione-sepharose 4B (GE Healthcare BioSciences Co., Piscataway, NJ, USA) to obtain GST-ERR-γ –LBD. The glutathione used for elution of GST-ERR-γ –LBD from the column was removed by gel filtration on a column of Sephadex G-10 (15 × 100 mm; GE Healthcare Bio-Sciences Co.) equilibrated with 50 mM Tris-HCl (pH 8.0), and the protein content (506.24 μg/mL) was estimated by the Bradford method using a Protein Assay CBB Solution (Nakarai Tesque). Preparation of GST-fused ER-α –LBD was carried out as described previously (Takayanagi et al. 2006).

### Radioligand binding assays for saturation binding

The saturation binding assay for GST-ERR-γ –LBD was conducted at 4°C using [^3^H]BPA (5 Ci/mmol; Moravek Biochemicals, Brea, CA, USA) with or without BPA (10 μM in the final solution). Purified protein (0.32 μg/mL) was incubated with increasing concentrations of [^3^H]BPA (2.1–24.3 nM) in a final volume of 100 μL of binding buffer [10 mM HEPES (pH 7.5), 50 mM sodium chloride, 2 mM magnesium chloride, 1 mM EDTA, 2 mM CHAPS {3-[(3-cholamidopropyl)dimethylammonio]-1-propanesulfonate}, and 2 mg/mL γ-globulin]. Nonspecific binding was determined in a parallel set of incubations that included 10 μM nonradiolabeled BPA. After incubation for 2 hr at 4°C, all the fractions were filtered by the direct vacuum filtration method (MultiScreen_HTS_ HV, 0.45 μm pore size; Millipore, Billerica, MA, USA) for the B/F separation (the separation of receptor-bound ligand from free ligand) (Nakai et al. 1999). Filtration was carried out on a multiscreen separation system (Millipore). Before filtration, 100 μL of 1% dextran-coated charcoal (DCC) (Sigma) in phosphate buffer (pH 7.4) was added to the assay vessels, and the mixture was incubated for 10 min on ice. The radioactivity of the filtered solution was counted on a liquid scintillation counter (LS6500; Beckman Coulter, Fullerton, CA, USA). The saturation assay was performed in triplicate. The specific binding of [^3^H]BPA was calculated by subtracting the nonspecific binding from the total binding.

### Radioligand binding assays for competitive binding

BPA and the BPA-related chemicals were dissolved in a binding buffer containing 0.3–1.0% *N*,*N*-dimethylsulfoxide (DMSO). These compounds were examined for their ability to inhibit the binding of [^3^H]BPA (3 nM in the final solution) to GST-ERR-γ –LBD (0.32 μg/mL in the final solution). The reaction mixtures were incubated for 2 hr at 4°C and free radioligand was removed with 1% DCC by filtration as described above. Radioactivity was determined on a liquid scintillation counter (TopCount NXT; PerkinElmer Life Sciences Tokyo, Japan). The IC_50_ values (the concentrations for the half-maximal inhibition) were calculated from the dose–response curves obtained using the nonlinear analysis program ALLFIT (De Lean et al. 1978). Each assay was performed in duplicate and repeated at least three times. The competitive binding assay for GST-ER-α –LBD was carried out as described above using [^3^H]E_2_ (5.74 TBq/mmol; Amersham Biosciences, Buckinghamshire, UK).

### Cell culture and transient transfection assays

HeLa cells were maintained in Eagle’s MEM (EMEM; Nissui, Tokyo, Japan) in the presence of 10% (vol/vol) fetal bovine serum at 37°C. For luciferase assays, HeLa cells were seeded at 5 × 10^5^ cells/6-cm dish for 24 hr and then transfected with 4 μg of reporter gene (pGL3/3 × ERRE) and 3 μg of ERR-γ expression plasmids (pcDNA3/ERR-γ ) by Lipofectamine Plus reagent (Invitrogen Japan, Tokyo, Japan) according to the manufacturer’s protocol. Approximately 24 hr after transfection, cells were harvested and plated into 96-well plates at 5 × 10^4^ cells/well. The cells were then treated with varying doses of chemicals diluted with 1% bovine serum albumin/phosphate-buffered saline (BSA/PBS, vol/vol). To measure the antagonistic activity, a fixed concentration of compounds (10^−5^ M to 10^−10^ M in the final solution) was added along with 4-OHT. After 24 hr, luciferase activity was measured with the appropriate reagent using a Luciferase Assay System (Promega, Madison, WI, USA) according to the manufacturer’s instructions. Light emission was measured using a Wallac 1420 ARVOsx multi-label counter (PerkinElmer). Cells treated with 1% BSA/PBS were used as a vehicle control. Each assay was performed in triplicate and repeated at least three times.

## Results and Discussion

### Highly specific binding of BPA to ERR-γ

To demonstrate the direct binding of BPA to ERR-γ , we first attempted to establish a saturation receptor binding assay using radio-labeled BPA. We analyzed the saturation binding of [^3^H]BPA against the recombinant ERR-γ –LBD protein, to which GST was fused at the N-terminus. In the actual receptor binding assay, we used [^3^H]BPA (2.0–24 nM) against purified protein at a concentration of 0.32 μg/mL, which corresponds to a concentration of 6.3 nM. The removal of receptor-free [^3^H]BPA was carried out with 1% DCC. In this procedure, DCC mixtures were transferred to a 96-well HV-plate with a filter (0.45-μm pore size) for direct vacuum.

**Figure 1:**
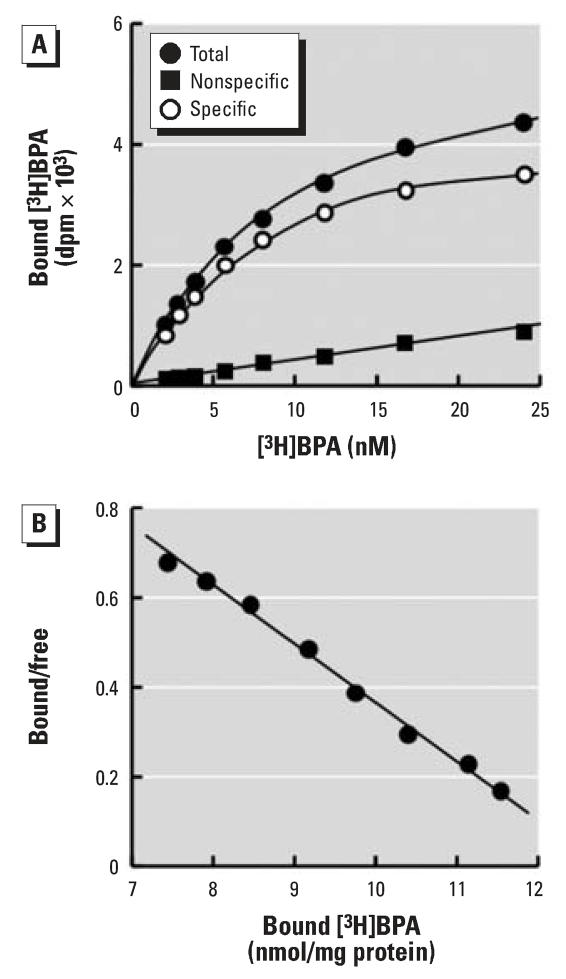
The saturation binding analysis of BPA for ERR-γ . (*A*) Saturation binding curve of [^3^H]BPA for the recombinant human ERR-γ –LBD showing total, nonspecific, and specific binding. Determination of nonspecific binding was carried out by excess unlabeled BPA (10 μM). (*B*) Binding data analyzed by Scatchard plot analysis to estimate the dissociation constant (*K*_D_) and the receptor density (*B*_max_). The plot was linear, the *K*_D_ value was estimated to be 5.50 ± 0.87 nM, and *B*_max_ was 14.4 nmol/mg protein. The saturation binding analysis was performed in duplicate and repeated four times.

As shown in Figure 1A, the binding of BPA to ERR-γ was specific and saturated. Specific binding of [^3^H]BPA to ERR-γ was estimated to be approximately 80%, which we judged to be a very high value. In other words, the level of nonspecific binding of [^3^H]BPA was very low (Figure 1A). The high level of specific binding of [^3^H]BPA clearly demonstrated that BPA has no structural elements for nonspecific binding to the receptor protein and exclusively occupies the binding pocket of ERR-γ –LBD. GST did not bind [^3^H]BPA at all. It should be noted that the specific binding of [^3^H]4-OHT was only about 50% (Takayanagi et al. 2006).

The Scatchard plot analysis showed a distinct single binding mode (Figure 1B). From the slope, the binding affinity constant (*K*_D_) was calculated to be 5.50 nM. The receptor density (*B*_max_) was estimated to be 14.4 nmol/mg protein, which is roughly compatible with the calculated value of 18.9 nmol/mg protein. The *B*_max_ value of [^3^H]4-OHT is much smaller than that of [^3^H]BPA. These results further demonstrate that ERR-γ binds [^3^H]BPA very specifically and exclusively.

### Binding ability of BPA to ERR-γ

We performed the competitive receptor binding assay using [^3^H]BPA (3 nM in the final solution) for GST–ERR-γ –LBD (0.32 μg/mL in the final solution). To confirm that BPA is a truly specific ligand for ERR-γ , we tested all nonradiolabeled BPA compounds available in Japan, which we obtained from seven different reagent companies. Because the compounds all had different levels of purity (95–99%), we adjusted their initial concentration, 1.0 × 10^−2^ M, based on the purity indicated on the label.

We found that BPA displaces [^3^H]BPA in a dose-dependent manner. Its binding curve was sigmoidal in a single binding mode (slope = ~ 1), which afforded an average IC_50_ value of 9.78 nM. We found all BPA compounds purchased to be equally potent. These results clearly demonstrate that BPA binds very strongly to the NR ERR-γ

### 4-OHT as a potent displacer of BPA in ERR-γ

4-OHT has been reported to potently displace [^3^H]4-OHT in the binding to ERR-γ (Greschik et al. 2004; Takayanagi et al. 2006). In the present study, 4-OHT very potently displaced [^3^H]BPA (IC_50_ = 10.9 nM) (Table 1). BPA and 4-OHT yielded sigmoidal binding curves indistinguishable from each other (data not shown), indicating that the two are almost equipotent. These results obtained using the [^3^H]BPA tracer were almost identical to those obtained by [^3^H]4-OHT (Takayanagi et al. 2006).

BPA and 4-OHT share only a phenol group, and thus the phenol groups of these compounds are highly likely to occupy the same binding site in the ERR-γ receptor. Because the phenol group of 4-OHT is anchored by hydrogen bonds to Glu275 and Arg316 of ERR-γ (Greschik et al. 2004), the phenol group of BPA may also bind to these ERR-γ residues. Indeed, this has been proven by our recent X-ray crystal structure analysis of the complex between BPA and human ERR-γ –LBD (Matsushima et al. 2007). Hereafter, we designate the benzene ring of this phenol group of BPA as the A-ring and the additional benzene ring as the B-ring.

### BPA-methyl as a structural requirement for binding to ERR-γ

**Figure 2:**
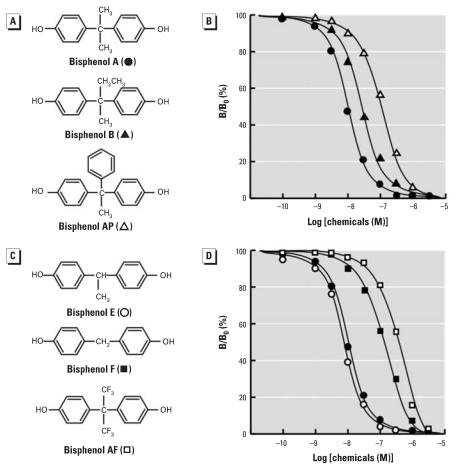
Chemical structure of BPA and its derivatives and their dose–response curves in the radioligand receptor binding assay for ERR-γ . (*A*) Chemical structures of BPA (two methyl groups) and its derivatives: bisphenol B (a methyl group and an ethyl group) and bisphenol AP (a methyl group and a phenyl group). (*B*) Binding activities of BPA, bisphenol B, and bisphenol AP examined by the competitive binding assay using [^3^H]BPA and GST–ERR-γ –LBD. (*C*) Chemical structures of bisphenol E (one methyl group) and its derivatives, bisphenol F and bisphenol AF [two trifluoromethyl groups (CF_3_)]. (*D*) Binding activities of BPA, bisphenol E, bisphenol F, and bisphenol AP examined by the competitive binding assay. (*B*) and (*D*) each show representative curves with the IC_50_ values closest to the mean IC_50_ from at least five independent assays for each compound. B/B_0_ is the relative inhibitory activity estimated from the calculation of the percentage of displacement by the chemical tested (B) against the specific binding (B_0_ = 100%) of [^3^H]BPA.

We evaluated the role of the two methyl (CH_3_) groups on the sp^3^-C atom of BPA in binding to ERR-γ by a series of analogs of BPA, HO-C_6_H_4_-C(CH_3_)_2_-C_6_H_4_-OH. First, we examined the effect of incorporation of the methyl group on the binding affinity of BPA. When CH_3_ was incorporated into the parent methyl group to produce HO-C_6_H_4_-C(CH_3_)(CH_2_CH_3_)-C_6_H_4_-OH (Figure 2A), we found the resulting bisphenol B to be approximately half as potent (IC_50_ = 26.3 nM) as BPA (Table 1). This result clearly indicates that a bulky group on the central sp^3^-C atom is obviously disadvantageous in terms of the binding of BPA to ERR-γ ’s binding pocket.

On the other hand, an enhancement of activity was observed when one of the methyl groups was eliminated from BPA. The resulting bisphenol E [HO-C_6_H_4_-CH(CH_3_)-C_6_H_4_-OH] (Figure 2C) exhibited slightly better binding activity (IC_50_ = 8.14 nM) than BPA (Table 1). Bisphenol E is indeed the most potent chemical to date for the NR ERR-γ (Figure 2D). The maximal activity was attained when one of the methyl groups was removed from BPA. Apparently, the concomitance of two methyl groups on the central sp^3^-C atom of BPA is disadvantageous and unfavorable.

The fact that a single methyl group had the best fit for ERR-γ was further demonstrated by the diminished activity of bisphenol AP, which has a phenyl group in place of the hydrogen atom that is found in bisphenol E (Figure 2A). Bisphenol AP exhibited approximately 15-fold weaker binding affinity for ERR-γ than bisphenol E, with IC_50_ = 123 nM (Figure 2B, Table 1). Steric hindrance by the benzene ring, as well as its electron-rich characteristics, might be responsible for this drop in the receptor binding affinity of bisphenol AP.

The importance of the remaining methyl group in bisphenol E became evident from the drastically reduced activity of bisphenol F [HO-C_6_H_4_-CH_2_-C_6_H_4_-OH]. This compound was approximately 16-fold less potent than bisphenol E, exhibiting an IC_50_ value of 131 nM (Table 1). All of these results clearly indicate that one of the two methyl groups is involved in the intermolecular interaction with the receptor residue(s). The interaction involving the CH_3_ group is a kind of hydrophobic interaction, such as CH_3_-alkyl and CH/π interactions.

The fundamental nature of this interaction involving the CH_3_ group became rather apparent from the binding result of bisphenol AF [HO-C_6_H_4_-C(CF_3_)_2_-C_6_H_4_-OH]. The CH_3_→ CF_3_ substitution in BPA creates this compound (Figure 2C), which has two electron-rich trifluoromethyl CF_3_ groups instead of the rather electron-poor methyl CH_3_ group. The molecular size of CF_3_ is almost equal to that of CH_3_. A drastically reduced activity of bisphenol AF, about 35-fold less potent (358 nM) than BPA (Table 1), thus demonstrates that the BPA’s CH_3_ group is in an electrostatic interaction with the electron-rich residue(s) of the receptor. Replacement of CH_3_ with CF_3_ is definitely disadvantageous, because CF_3_ is very electron-rich and thus brings about a strong repulsion with such electron-rich residues of the receptor. One of the electron-rich candidates of the receptor is the aromatic ring of Phe, Tyr, His, and Trp. Based on the reported X-ray crystal structure of ERR-γ , feasible candidates are Phe-435 and Phe-450 (Greschik et al. 2002, 2004; Matsushima et al. 2007; Wang et al. 2006).

### A single phenol-hydroxyl group is enough for BPA to bind to ERR-γ

**Figure 3:**
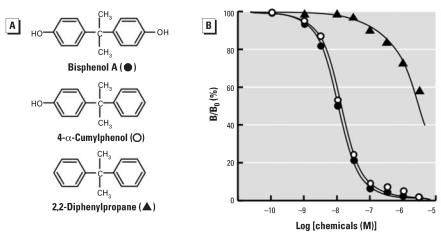
Chemical structure of BPA and its derivatives lacking the hydroxyl group(s) and their dose–response curves in the radioligand receptor binding assay for ERR-γ . (*A*) Chemical structure of BPA and its derivatives lacking the hydroxyl group(s): 4-α-cumylphenol (without one hydroxyl group from BPA), and 2,2-diphenylpropane (without either hydroxyl groups from BPA). (*B*) Binding activities of BPA, 4-α-cumylphenol, and 2,2-diphenylpropane examined by the competitive binding assay using [^3^H]BPA and GST-ERR-γ –LBD; representative curves indicate the IC_50_ value closest to the mean IC_50_ from at least five independent assays for each compound.

BPA has a very simple symmetrical chemical structure of HO-C_6_H_4_-C(CH_3_)_2_-C_6_H_4_-OH (Figure 2A). When one of the phenol-hydroxyl groups (–OH) of BPA was eliminated, the resulting 4-α-cumylphenol (HO-C_6_H_4_-C(CH_3_)_2_-C_6_H_5_; Figure 3A) still bound very strongly to ERR-γ . 4-α-Cumylphenol was as potent as BPA (Figure 3B), having an IC_50_ value of 10.6 nM (Table 2). Contrary to the expectation that both of the phenol-hydroxyl groups of BPA would participate in the hydrogen bonds, this result indicates that the second hydroxyl group does not necessarily participate in the hydrogen bonding. Given that this hydroxyl group forms a hydrogen bond with the ERR-γ receptor residue, the bond would be considered extremely weak, as suggested by the X-ray crystal analysis of 4-α-cumylphenol–ERR-γ complex (Matsushima A, Teramoto T, Okada H, Liu X, Tokunaga T, Kakuta Y, Shimohigashi Y, unpublished data).

When both of the phenol-hydroxyl groups were eliminated from BPA, the resulting 2,2-diphenyl propane [C_6_H_5_-C(CH_3_)_2_-C_6_H_5_] was almost completely inactive (Figure 3B, Table 2). This compound elicits only about 30% inhibition of the binding of [^3^H]BPA at the 1-μM concentration, whereas BPA almost completely inhibits the binding of [^3^H]BPA at this concentration (Figure 3B). It is clear that one of the phenol-hydroxyl groups of BPA is indispensable for the interaction with a binding pocket of ERR-γ . These results, together with the fact that 4-α-cumylphenol and BPA are equipotent, emphasizes the significance of one of the two phenol groups in the interaction of BPA with ERR-γ . As described above, this hydroxyl group should be attached to the benzene A-ring. It became apparent that the phenol-hydroxyl group attached to another phenol-benzene ring (B-ring) is not necessarily required for binding of BPA to ERR-γ .

### BPA-phenol as a structural requirement for binding to ERR-γ

**Figure 4:**
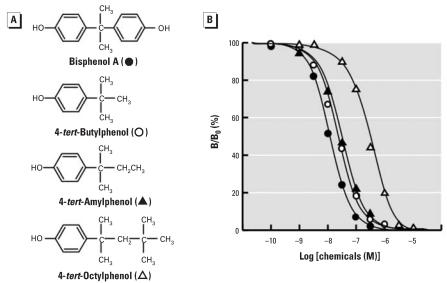
Chemical structure of BPA and its derivatives lacking the phenol group and their dose–response curves in the radioligand receptor binding assay for ERR-γ . (*A*) Chemical structure of BPA and its derivatives with the alkyl group at the position of phenol group: 4-*tert*-butylphenol (a methyl group); 4-*tert*-amylphenol (an ethyl group); and 4-*tert*-octylphenol (a *tert*-butyl methyl group). (*B*) Binding activities of BPA, 4-*tert*-butylphenol, 4-*tert*-amylphenol, and 4-*tert*-octylphenol examined by the competitive binding assay using [^3^H]BPA and GST-ERR-γ –LBD; representative curves indicate the IC_50_ value closest to the mean IC_50_ from at least five independent assays for each compound.

As described above, 4-α-cumylphenol is as active as BPA. The importance of the benzene B-ring can be examined by replacing the B-ring with the alkyl groups. When the benzene B-ring of 4-α-cumylphenol was substituted with either methyl or ethyl, the resulting 4-*tert*-butylphenol [HO-C_6_H_4_-C(CH_3_)_2_-CH_3_] and 4-*tert*-amylphenol [HO-C_6_H_4_-C(CH_3_)_2_-CH_2_CH_3_] (Figure 4A) were considerably potent (Figure 4B), with values of 26.1 nM and 33.2 nM, respectively (Table 2). This reveals that alkyl groups can be substituted for the aromatic benzene ring without affecting the basal binding capability.

However, because both 4-*tert*-butylphenol and 4-*tert*-amylphenol are still a few times less active than 4-α-cumylphenol, a specific binding site of ERR-γ appears to prefer the aromatic benzene ring to the alkyl groups. This suggests that BPA’s second phenol-phenyl group (benzene B-ring) is in the π interaction with the receptor residue(s), that is, either a XH/π interaction (X = N, O, and C) or a π /π interaction. The most plausible candidate for the receptor residue in this interaction is the Tyr residue at position 326 of ERR-γ . Indeed, the phenol-hydroxyl group of this Tyr-326 was found in the OH/π interaction with the B-ring of BPA (Matsushima et al. 2007).

**Figure 5:**
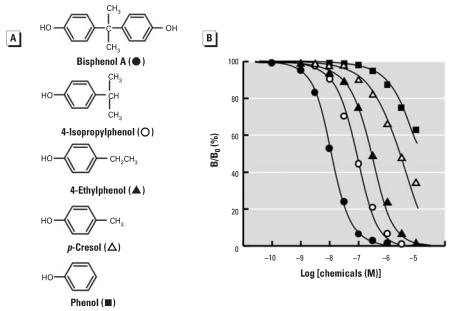
Chemical structure of BPA and a series of alkyl phenols and their dose–response curves in the radioligand receptor binding assay for ERR-γ . (*A*) Chemical structure of BPA and its derivatives with the alkyl group at the *para* position: 4-isopropylphenol (a 4-isopropyl group); 4-ethylphenol (an ethyl group); *p*-cresol (a methyl group); and phenol (a hydrogen atom). (*B*) Binding activities of BPA, 4-isopropylphenol, 4-ethylphenol, *p*-cresol, and phenol examined by the competitive binding assay using [^3^H]BPA and GST-ERR-γ –LBD; representative curves indicate the IC_50_ value closest to the mean IC_50_ from at least five independent assays for each compound.

In a BPA molecule, two C_6_H_4_-OH (phenol) groups are connected to the sp^3^ carbon atom (sp^3^-C) together with two CH_3_ (methyl) groups. The most simple structure–activity study is to compare the activity of compounds lacking one of these groups. The compound that lacks the phenol group is 4-isopropylphenol [HO-C_6_H_4_-CH(CH_3_)_2_] (Figure 5A), and this *para*-isopropyl phenol was fairly potent at displacing [^3^H]BPA (Figure 5B), with an IC_50_ value of 71.1 nM (Table 2). However, 4-isopropylphenol was still approximately 7-fold less active than BPA, indicating that the phenol backbone structure is an essential structural element for the binding to ERR-γ.

When one of the two methyl groups was eliminated from 4-isopropylphenol, the resulting 4-ethylphenol [HO-C_6_H_4_-CH_2_-CH_3_] (Figure 5A) was found to be very weakly active (289 nM) (Table 2). Elimination of another methyl group still afforded a compound of inactive *p*-cresol [HO-C_6_H_4_-CH_3_], but with the IC_50_ value being approximately 1.3 μM. Phenol [HO-C_6_H_5_] tended to bind to ERR-γ (Figure 5B). These results clearly indicate that the phenol group is a core structure for the attachment of BPA to ERR-γ.

### 4-Alkyl phenols as putative potent binders to ERR-γ

Attachment of the methyl group to 4-isopropylphenol [HO-C_6_H_4_-CH(CH_3_)_2_] to create 4-*tert*-butylphenol [HO-C_6_H_4_-C(CH_3_)_3_] considerably facilitates the binding of the phenol derivative to ERR-γ (Table 2). 4-*tert*-Amylphenol [HO-C_6_H_4_-C(CH_3_)_2_-CH_2_CH_3_] is almost as active as 4-*tert*-butylphenol. However, 4-*tert*-octylphenol [HO-C _6_ H _4_ -C(CH _3_ ) _2_ -CH _2_ -C(CH _3_ ) _3_ ] (Figure 4B) was significantly weaker (approximately 10 times less potent) than 4-*tert*-butylphenol (Table 2). Thus, the activities of H O - C _6_ H _4_ - C ( C H _3_ ) _2_ - C H ( C H _3_ ) _2_ , HO-C_6_H_4_-C(CH_3_)_2_-CH(CH_3_)_3_, HO-C_6_H_4_-C(CH_3_)_2_-CH_2_-CH_2_-CH_3_, and HO-C_6_H_4_-C(CH_3_)_2_-CH_2_-CH(CH_3_)_2_ are expected to be intermediate between those of 4-*tert*-amyl-phenol and 4-*tert*-octylphenol, although these molecules are not commercially available. It appears that, among the 4-alkylphenols of HO-C_6_H_4_-C(CH_3_)_2_-C_n_H_2n+1_ (=R), 4-*tert*-butylphenol (R = CH_3_) and 4-*tert*-amylphenol (R = CH_2_-CH_3_) show the maximum competitive activity with the binding of ERR-γ.

The structural comparison of HO-C_6_H_4_-C(CH_3_)_2_-CH_3_ (4-*tert*-butylphenol), HOC_6_H_4_-C(CH_3_)_2_-CH_2_CH_3_ (4-*tert*-amylphenol), and BPA HO-C_6_H_4_-C(CH_3_)_2_-C_6_H_4_-OH clearly indicated that the R group should not be bulky for high receptor binding activity. A plain π electron-rich benzene aromatic ring is thus optimal for interaction with the receptor residue of ERR-γ-Tyr326.

### Inhibitory activity of BPA derivatives for ERR-γ

**Figure 6:**
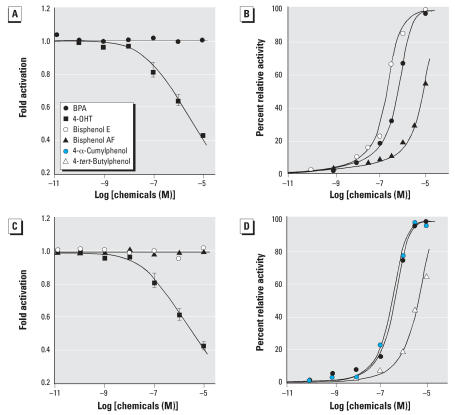
Luciferase-reporter gene assay of BPA and its derivatives for human ERR-γ . (*A*) Deactivation of the fully activated human ERR-γ by the inverse agonist 4-OHT and sustainment by BPA. (*B*) Reversing activity of BPA, bisphenol E, and bisphenol AF against the inverse agonist activity of 1.0 μM 4-OHT; 1.0 μM 4-OHT exhibited approximately 0.4-fold deactivation, and the inhibitory activities are shown by the percentage of relative activity. (*C*) Sustainment of the fully activated human ERR-γ by bisphenol E and bisphenol AF together with inverse agonist activity by 4-OHT. (*D*) Reversing activity of BPA, 4-α-cumylphenol, and 4-*tert*-butylphenol; the inverse agonist activity of 4-OHT was clearly reversed by all bisphenols tested in a dose-dependent manner. Data are from a single experiment performed in triplicate; two additional experiments gave similar results. High basal constitutive activity of ERR-γ was evaluated with the luciferase-reporter plasmid (pGL3/3 × ERRE), and the highest activity was estimated in a cell preparation of 1.0 × 10^5^ HeLa cells/well.

We found that BPA retained a high constitutive basal activity of ERR-γ in the luciferase reporter gene assay (Figure 6A). ERR-γ is in a full activation with no ligand; it is one of the self-activated NRs and is deactivated by the so-called “inverse agonists” such as 4-OHT (Greschik et al. 2004; Takayanagi et al. 2006). Although BPA shows no apparent effect on the high basal activity of ERR-γ , BPA evidently antagonizes or inhibits the deactivation activity of 4-OHT in a dose-dependent manner (Figure 6B), as reported by Takayanagi et al. (2006). This neutral antagonist is a distinct inhibitor or suppressor of the inverse agonist, reversing the deactivation conformation to the activation conformation.

All of the potent BPA derivatives (i.e., bisphenol E, bisphenol AF, 4-α-cumylphenol, and 4-*tert*-butylphenol) were found, just like BPA, to retain a high constitutive basal activity of ERR-γ in the same luciferase reporter gene assay (Figure 6C). In addition, these compounds inhibited the inverse agonist activity of 4-OHT and thus were specific inhibitors against the inverse agonist 4-OHT. Their abilities to antagonize 4-OHT are approximately one order lower than their binding potencies to ERR-γ (Figure 6B,D). This discrepancy is probably caused by the inclusion of a number of co-effecter proteins for eliciting a gene expression in the luciferase reporter gene assay.

### Receptor selectivity of BPA derivatives for ERR-γ over ER-α

We classified BPA and its derivatives into the four groups, depending on their receptor binding affinity for ERR-γ : that is, group A, BPA and chemicals as potent as BPA; group B, chemicals considerably potent; group C, chemicals moderately potent; and group D, inactive chemicals. All chemicals were then examined for their ability to bind to ER-α , and the affinity measured was compared respectively with that for ERR-γ (Table 3). As reported previously (Takayanagi et al. 2006), BPA is highly selective for ERR-γ . It binds to ER-α only weakly; we calculated BPA’s receptor selectivity to be 105, which suggests that BPA prefers ERR-γ 105 times more strongly than ER-α . Other group A compounds, namely, bisphenol E and 4-α-cumylphenol, were also greatly selective for ERR-γ (Table 3). In particular, bisphenol E was found to be exclusively selective and specific for ERR-γ because it was almost completely inactive for ER-α.

*para*-Alkyl phenols in group B (IC_50_*^ERR-^*^γ^ = of 26–71 nM) were also almost completely inactive for ER-α (Table 3). Those include 4-*tert*-butylphenol, 4-*tert*-amylphenol, and 4-isopropylphenol, and they were fully selective and specific for ERR-γ. In contrast, bisphenol B was very weakly active (246 nM) for ER-α, although it was still selective (about 9.5 times) for ERR-γ.

Among group C chemicals (IC_50_*^ERR-^*^γ^ = 120–350 nM), bisphenol F was almost completely inactive for ER-α , making it fully selective for ERR-γ (Table 3). This was also true for 4-ethylphenol. Bisphenol AP showed a weak binding affinity (361 nM) for ER-α , but it was still selective (about 3 times) for ERR-γ . However, bisphenol AF emerged as a ligand selective for ER-α with a selectivity ratio of 0.15 (Table 3). The reciprocal of 0.15 [i.e., ERR-γ (IC_50_)/ER-α (IC_50_) = 6.67] denotes a selectivity ratio of bisphenol AF for ER-α.

The results clearly indicate that the alkyl groups on the central sp^3^-C atom of bisphenol derivatives play a key role in selection of the NR ERR-γ and ER-α . When we checked the receptor binding activities of one series of bisphenol derivatives (i.e., bisphenol E, BPA, bisphenol B, bisphenol AP, and bisphenol AF), we found this line-up to be the order of compounds with increasing affinity to ER-α . At the same time, it was the order of compounds with decreasing affinity to ERR-γ . ERR-γ prefers the less bulky and less electrophilic alkyl groups, whereas ER-α appears to prefer the bulkier and more electrophilic alkyl groups.

4-*tert*-Octylphenol is a well-known endocrine disruptor candidate, but it was only moderately potent for ERR-γ (IC_50_ = 238 nM; Table 2). However, it was considerably weak for ER-α , with an IC_50_ of 925 nM; thus, we judged 4-*tert*-octylphenol to be somewhat selective (approximately 4 times) for ERR-γ . Another representative endocrine disruptor candidate is 4-nonylphenol, which was moderately active for ERR-γ (Takayanagi et al. 2006). Thus, 4-nonylphenol was slightly more selective for ERR-γ . However, some 4-alkyl phenols are distinctly more potent for ERR-γ than 4-*tert*-octylphenol and 4-nonylphenol: 4-*tert*-butylphenol, 4-*tert*-amylphenol, and 4-isopropylphenol. These 4-alkyl phenols are definitely novel candidates of the endocrine disruptor specific for ERR-γ.

## Conclusion

In the present study we have shown that all the structural elements of BPA—the phenol and methyl groups and the phenyl group on the central sp^3^-C atom—are prerequisite for binding to the NR ERR-γ . Furthermore, we have shown that the phenol derivatives are potent candidates for the endocrine disruptor that binds to ERR-γ . The binding affinity of [^3^H]BPA to ERR-γ –LBD is extremely high, with a *K*_D_ value of 5.50 nM. Thus, it appears to be important to evaluate whether the previously reported effects of BPA at low doses are mediated through ERR-γ and its specific target gene(s).

At the same time, it is necessary to clarify the physiologic roles of ERR-γ and to examine the degree and ways in which BPA may influence these. This is particularly important because ERR-γ is expressed in a tissue-restricted manner—for example, it is expressed very strongly in the mammalian fetal brain and placenta—at sites that could have important outcomes for newborns. Recently, many lines of evidence have indicated that low doses of BPA affects the central nervous system (reviewed by vom Saal and Welshons 2005; Welshons et al. 2003, 2006). The molecular mechanism for these effects could involve, at least in part, the high affinity binding of BPA to ERR-γ . A similar phenomenon may be observed for other NRs, and the exploration of such chemical–receptor interactions requires a specific assay system or concept applicable to all the NRs.

## Figures and Tables

**Table 1:** Receptor binding affinity (mean ± SE) of BPA and its analogs, and 4-OHT for ERR-γ.

Chemical	Binding affinity (IC_50_, nM)
BPA	9.78 ± 0.87
Bisphenol AF	358 ± 30.5
Bisphenol AP	123 ± 15.1
Bisphenol B	26.3 ± 2.65
Bisphenol E	8.14 ± 0.83
Bisphenol F	131 ± 17.9
4-OHT	10.9 ± 0.91

**Table 2:** The receptor binding affinity (mean ± SE) of BPA and its derivatives lacking of the phenol group for human ERR-γ .

Chemical	Binding affinity (IC_50_, nM)
BPA	9.78 ± 0.87
4-α-Cumylphenol	10.6 ± 0.87
2,2-Diphenylpropane	ND
4-*tert*-Butylphenol	26.1 ± 2.45
4-*tert*-Amylphenol	33.2 ± 2.85
4-Isopropylphenol	71.1 ± 7.73
4-*tert*-Octylphenol	238 ± 28.1
4-Ethylphenol	289 ± 45.9
*p*-Cresol	1,290 ± 72.5
Phenol	ND

ND, not determined (IC_50_ value could not be calculated because of extremely weak binding activity, even at a 10 μM concentration).

**Table 3:** Receptor binding affinity (mean ± SE; *n* = 3) of BPA and its analogs for ER-α and their receptor selectivity for ERR-γ over ER-α .

Chemical	Binding affinity for ER-α (IC_50_, nM)	ERR-γ receptor selectivity ER-α (IC_50_, nM)/ERR-γ (IC_50_, nM)
E_2_	0.88 ± 0.13	Exclusively ER-α
Group A (chemicals as active as BPA for ERR-γ )		
Bisphenol E	ND	Exclusively ERR-γ
BPA	1,030 ± 146	105
4-α-Cumylphenol	4,770 ± 510	450
Group B (chemicals considerably potent for ERR-γ )		
Bisphenol B	246 ±29.7	9.46
4-*tert*-Butylphenol	ND	Exclusively ERR-γ
4-*tert*-Amylphenol	ND	Exclusively ERR-γ
4-Isopropylphenol	ND	Exclusively ERR-γ
Group C (chemicals moderately potent for ERR-γ )		
Bisphenol AP	361 ± 22.6	2.93
Bisphenol F	ND	Exclusively ERR-γ
4-*tert*-Octylphenol	925 ± 83.9	3.89
4-Ethylphenol	ND	Exclusively ERR-γ
Bisphenol AF	53.4 ± 7.28	0.15
Group D (chemicals extremely weak or inactive for ERR-γ)		
2,2-Diphenylpropane	ND	Inactive for both receptors
*p*-Cresol	ND	Almost inactive for both receptors
Phenol	ND	Inactive for both receptors

ND, not determined (IC_50_ value could not be calculated because of extremely weak binding activity even at a 10-μM concentration).
